# Maternal vitamin D deficiency impairs Treg and Breg responses in offspring mice and deteriorates allergic airway inflammation

**DOI:** 10.1186/s13223-020-00487-1

**Published:** 2020-10-14

**Authors:** Fei Huang, Yang-hua Ju, Hong-bo Wang, Ya-nan Li

**Affiliations:** 1 Department of Orthopedics, China-Japan Union Hospital of Jilin University, Changchun, 130033 Jilin China; 2 Department of Pediatrics, the First Hospital of Jilin University, Changchun, 130021 Jilin China; 3 Institute of Pediatrics, the First Hospital of Jilin University, Changchun, 130021 Jilin China

**Keywords:** Vitamin D deficiency, Pregnant, Offspring, Regulatory T cells, Regulatory B cells, Asthma

## Abstract

**Background:**

Vitamin D (VitD) can regulate immune responses and maternal VitD-deficiency can affect immune responses in the offspring. This study aimed at investigating the effects of maternal VitD-deficiency during pregnancy on Treg and Breg responses in offspring mice with house dust mite (HDM)-induced allergic airway inflammation.

**Methods:**

Female BALB/c mice were randomized and fed with normal chow or VitD-deficient diet until their offspring weaned. The offspring mice were fed with normal chow and injected with vehicle or HDM to induce allergic airway inflammation. The levels of serum 25(OH)D, cytokines and infiltrate numbers as well as percentages of Tregs and Bregs in the bronchoalveolar lavage fluid (BALF) were analyzed. The relative levels of VitD receptor (VDR), VitD-binding protein (VDBP), Cytochromes P450 (CYP) 27b1, and CYP24A1 mRNA transcripts in the lungs of different groups of mice were measured.

**Results:**

Maternal VitD-deficiency significantly reduced serum 25(OH)D levels in offspring mice. VitD-deficiency significantly increased the relative levels of VDR, VDBP and CYP27B1 mRNA transcripts, but decreased CYP24A1 expression in the lungs of mice. In comparison with the control mice, significantly elevated levels of pro-inflammatory cytokines, increased numbers of lymphocytes and eosinophils, but decreased levels of anti-inflammatory cytokines were detected in the BALF of VitD-deficient mice. VitD-deficiency significantly increased the frequency of Th1, Th2, Th9, Th17 cells, but decreased regulatory T (Tregs) and B cells (Bregs) in the BALF of mice with allergic airway inflammation.

**Conclusion:**

Maternal VitD-deficiency lowed serum 25(OH)D levels and enhanced HDM-induced allergic airway inflammation in the offspring by impairing Breg and Treg responses.

## Background

Asthma is a chronic disease and can affect million people yearly in the world, particularly for children. Pathologically, asthma is characterized by airway epithelial inflammation with different types of inflammatory infiltrates. These inflammatory infiltrates can secrete cytokines to damage the airway epithelium of the lung, leading to hard breath and hypoxia [1]. However, the molecular pathogenesis of allergic airway inflammation remains unclear. Previous studies have shown that allergen-specific inflammatory Th2, Th17 and Th1 cells are crucial for the pathogenesis of allergic airway inflammation [2] and deficiency in anti-inflammatory regulatory T (Tregs) and B cells (Bregs) also contributes to the development and deterioration of allergic airway inflammation. Actually, Tregs and Bregs can inhibit the pathogenic process of airway epithelial inflammation in rodents [3, 4]. Hence, further understanding of the pathogenic process and its regulators will be of significance in discovering new therapeutic targets for intervention of allergic airway inflammation in humans.

A growing number of clinical studies have shown that maternal vitamin D (VitD) deficiency in women significantly increases the risk of allergic airway inflammation in later life of their offspring [5]. A recent study has shown a global epidemic of VitD deficiency in pregnant women and about 84% of pregnant women have lower serum VitD concentrations [6]. Maternal 25-(OH)D can be transported to the fetus via the placenta during pregnancy and is the primary source of VitD to determine the levels of 25-(OH)D in umbilical cord blood of the fetus and newborn [7–9]. Previous studies have shown that maternal VitD deficiency in women may impair Treg responses. However, it is unclear whether the maternal VitD deficiency can impair Treg and Breg responses, and affect the expression of VitD regulators of VDR, VDBP, CYP24A1 and CYP27B1 in the offspring.

This study aimed to determine how maternal VitD deficiency affected serum 25-(OH)D levels in the offspring, allergic airway inflammation, Treg and Breg responses as well as the expression of VitD regulators of VDR, VDBP, CYP24A1 and CYP27B1 in mice following HDM-induced allergic airway inflammation.

## Materials and methods

### A mouse model of allergic airway inflammation

This study was conducted, according to the Animal Research and Care guidelines of the National Institutes of Health (NIH) and was approved by the Ethics Committee of Jilin University. Female BALB/c mice at 4–6 weeks of age (18–20 g) were obtained from the Animal Research Center of Jilin University, China, and housed in a specific pathogen-free facility with free access to food and water.

**Fig. 1:**
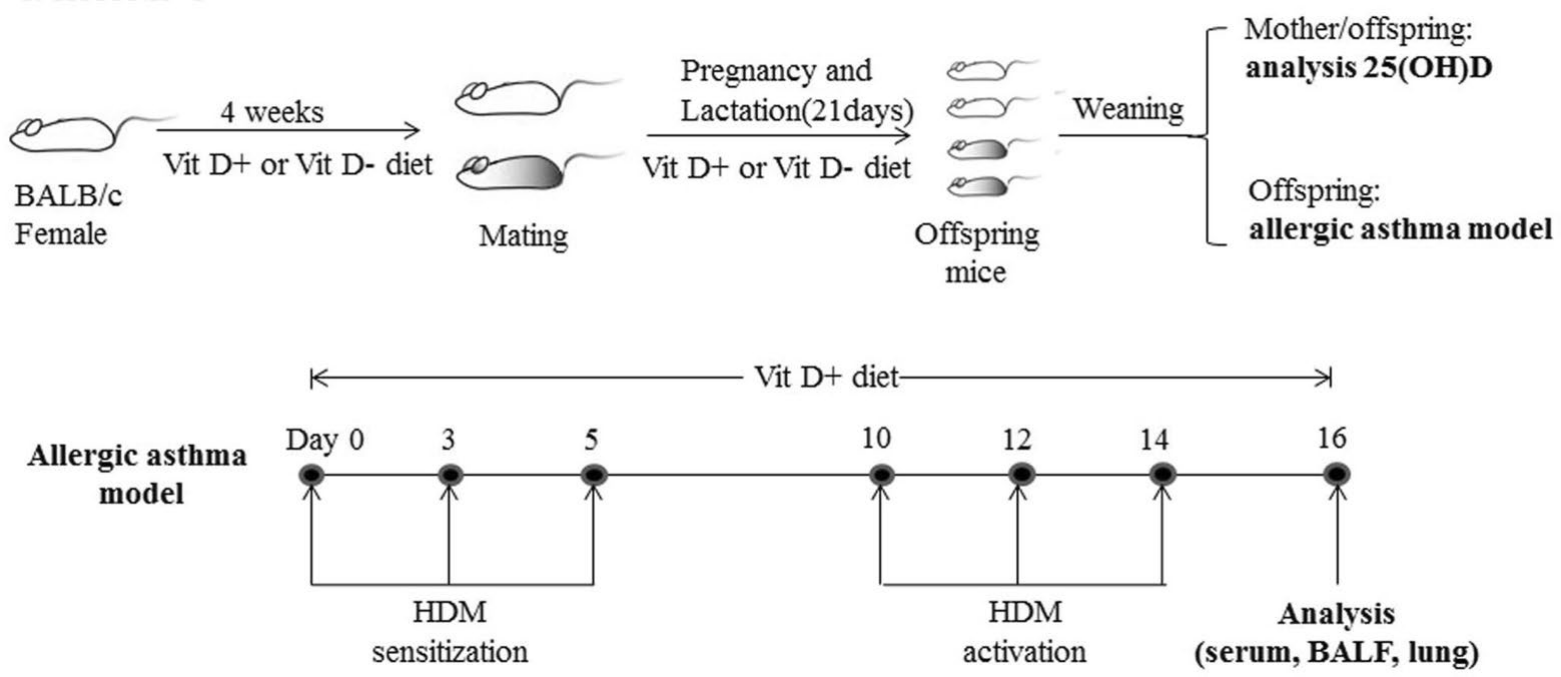
The illustration of generating VitD deficient mice and inducing allergic airway inflammation in mice by HDM

We established VitD deficient mice and a mouse model of allergic airway inflammation in the offspring, according to the protocol in Fig. 1. Briefly, the mice were randomized (20 mice per group) and fed with normal chow (1000 IU VitD3/kg) as the control or VitD deficient diet (lower than 25 IU VitD3/kg) as the (VitD-) for 4 weeks before mating, throughout the gestational period and up to weaning (21 days after birth). Subsequently, all of the mice were fed with normal chow. The mother mice were sacrificed and their serum samples were prepared. Three days after weaning (day 0), ten mice from each group were subjected to induction of allergic airway inflammation using HDM, as reported previously [10, 11]. Individual mice were injected intraperitoneally with 6 µg HDM in 30 µL PBS (PBS) at days 0, 3, and 5 for sensitization. The mice were injected with the same dose of HDM at days 10, 12 and 14 for activation of airway inflamamtion. The remaining 10 mice in each group were injected with the same volume of PBS and served as the PBS (Vit D−) and PBS (Vit D +) controls. Two days after the final injection, their peripheral venous blood samples were collected and serum samples were prepared, followed by stored at − 80 °C. The mice were sacrificed and they were subjected to the procedure of bronchoalveolar lavage (BAL). In addition, the lung tissues from individual mice were dissected and immediately frozen − 80 °C for homogenization and total RNA extraction.

### BAL, cytology, and cytokine measurements

Individual mice were catheterized in their trachea and their lungs were lavaged with 1 mL of ice-cold PBS containing EDTA and protease inhibitors for twice, followed by centrifuging BAL fluids. The cell number in the BALF samples was evaluated using a hemocytometer after Tuerk solution staining (Sigma-Aldrich, St. Louis, MO, USA). After cytospin, the cells were stained with May-Grünwald-Giemsa (Merck Millipore, Billerica, MA, USA) and the numbers of eosinophils, neutrophils, lymphocytes, and macrophages in the BALF samples were counted in a blinded manner. The levels of interleukin (IL)-4, IL-5, IL-13, IL-9, IL-10, IL-17A, IL-35, Interferon (IFN)-γ and tumor growth factor (TGF)-β1 in individual BALF samples were measured by enzyme-linked immunosorbent assay (ELISA) using specific kits (eBioscience, San Diego, CA, USA), according to the manufacturer’s instructions.

### Flow cytometry

The frequency of Tregs (CD4^+^CD25^+^Foxp3^+^), Bregs (CD19^+^IL-10^+^) cells, Th1 (CD3^+^CD8^−^IFN-γ^+^), Th2 (CD3^+^CD8^−^IL-4^+^), Th9 (CD3^+^CD8^−^IL-9^+^), and Th17 (CD3^+^CD8^−^IL-17A^+^) in individual BALF samples was determined by flow cytometry using fluorescent antibodies (BD Biosciences, San Jose, CA, USA) and a specific software provided. Briefly, the cells from individual BALF samples were stimulated with ionomycin (1 mg/mL; Sigma-Aldrich), PMA (25 ng/mL; Sigma-Aldrich) and monensin (2 nmol/mL) at 37 °C for 4 h. Subsequently, the cells were re-suspended in staining buffer and stained with Allophycocyanin (APC)-conjugated anti-CD3 (BD Biosciences, 565,643), Fluorescein isothiocyanate (FITC)-conjugated anti-CD8 (BD Biosciences, 553,031), followed by intracellularly-stained with phycoerythrin (PE)-anti-IFN-γ (BD Biosciences, 554,412), PE-anti-IL-4 (BD Biosciences, 554,389), PE-anti-IL-9 (BD Biosciences, 561,463), or PE-anti-IL-17A (BD Biosciences, 559,502), respectively. The control cells stained with a single antibody. After being washed, the cells were characterized by flow cytometry on a C6 Accuri flow cytometer (BD Biosciences). The cells were gated on living mononuclear cells and then on CD3 + CD8- cells. According to the single antibody staining, the frequency of Th1 (CD3^+^CD8^−^IFN-γ^+^), Th2 (CD3^+^CD8^−^IL-4^+^), Th9 (CD3^+^CD8^−^IL-9^+^), and Th17 (CD3^+^CD8^−^IL-17A^+^) cells in total CD3^+^CD8^−^ cells was analyzed. To measure the frequency of Tregs, the cells from each BALF sample were stained with APC-conjugated anti-CD4 (BD Biosciences, 561,091), FITC-conjugated anti-CD25 (BD Biosciences, 558,689). After fixed and permeabilized, the cells were, intracellularly-stained with PE-conjugated anti-Foxp3 (BD Biosciences, 563,101). The control cells were stained with a single antibody. The cells were gated on living mononuclear cells and then on CD4^+^CD25^+^ cells. According to the single antibody-stained control cells, the frequency of CD4^+^CD25^+^Foxp3^+^ cells in total CD4^+^CD25^+^ cells in individual samples was analyzed.

To determine the frequency of IL-10^+^ Bregs, BALF cells were stained with FITC-conjugated anti-CD19 (BD Biosciences, 553,785) and then stimulated with PMA, ionomycin and monensin at 37 °C for 4 h. Subsequently, the cells were intracellularly stained with PE-conjugated anti-IL-10 (BD Biosciences, 554,467). Control cells were stained with a single antibody. The stained cells were gated on living mononuclear cells, then on CD19^+^ cells. Based on the positivity of anti-IL-10 staining, the frequency of CD19^+^IL-10^+^ Bregs in individual samples was determined.

### Measurement of 25(OH)D

The levels of serum 25-(OH)D in individual mice were quantified by radioimmunoassay (RIA) using ^125^I-labelled 25(OH)D and a specific kit (DiaSorin, Stillwater, MN, USA), according to the manufacturer's instructions.

### Quantitative real-time PCR

We extracted total RNA from individual lung tissue samples using TRIzol Reagent (Invitrogen, Thermo Scientific, Waltham, MA, USA) and reversely transcribed 1.5 μg RNA into cDNA using the RevertAid First Strand cDNA Synthesis Kit (Thermo Scientific). After qualification and quantification of cDNA, we measured the relative levels of VDR, VDBP, CYP24a1 and CYP27b1 to the control Glyceraldehyde 3-phosphate dehydrogenase (GAPDH) by quantitative RT-PCR using the SYBR-Green PCR reaction system (iQ™ SYBR^®^ Green, Bio-Rad, Hercules, CA. USA) and specific primers. The sequences of primers were sense, 5′-CCTCTGGAAAGCTGTGGCGTG-3′; antisense, 5′-GCCAGTGAGCTTCCCGTTCAG-3′ for GAPDH (119 bp); sense, 5′-TCCCCCATCCCTAGAACCAG-3′; antisense, 5′-TCCTCATTGCTTGGGCTCTG-3′ for VDR (83 bp); sense, 5′-GGGAAACAGCAAGCCCAAAG-3′; antisense, 5′-GTAGCGTGAAAGCAGGGACC-3′ for VDBP (96 bp); sense, 5′-CCCTTCTGCAAGAAAACTGC-3′; antisense, 5′-CTCTTGAGGGCTCTGA TTGG-3′ for CYP24a1 (99 bp) and sense, 5′-GGTTCTCCGGAGCTTGTCTG-3′; antisense, 5′-AAACTGTGCGAAGTGTCCCA-3′ for CYP27b1 (164 bp) from Sangon Biotech (Shanghai, China). The data were normalized to GAPDH and analyzed by the 2^−∆∆Ct^ method.

### Statistical analysis

Statistical analyses were performed with SPSS version 24.0 for Windows (SPSS, Chicago, IL, USA) or GraphPad Prism 6 software (GraphPad Software, San Diego, CA, USA). Data are present as mean ± SD. The difference between groups was analyzed by Student T-test or Fisher exact test where applicable. The difference among groups was analyzed by ANOVA and post hoc Bonferroni test. Statistical significance was defined when a *p-value of* < 0.05.

## Results

### Feeding with low VitD3 diet in mothers lowers serum VitD3 levels, but increases eosinophil and lymphocyte infiltrates in the lung of their offspring following inducing allergic airway inflammation

**Fig. 2:**
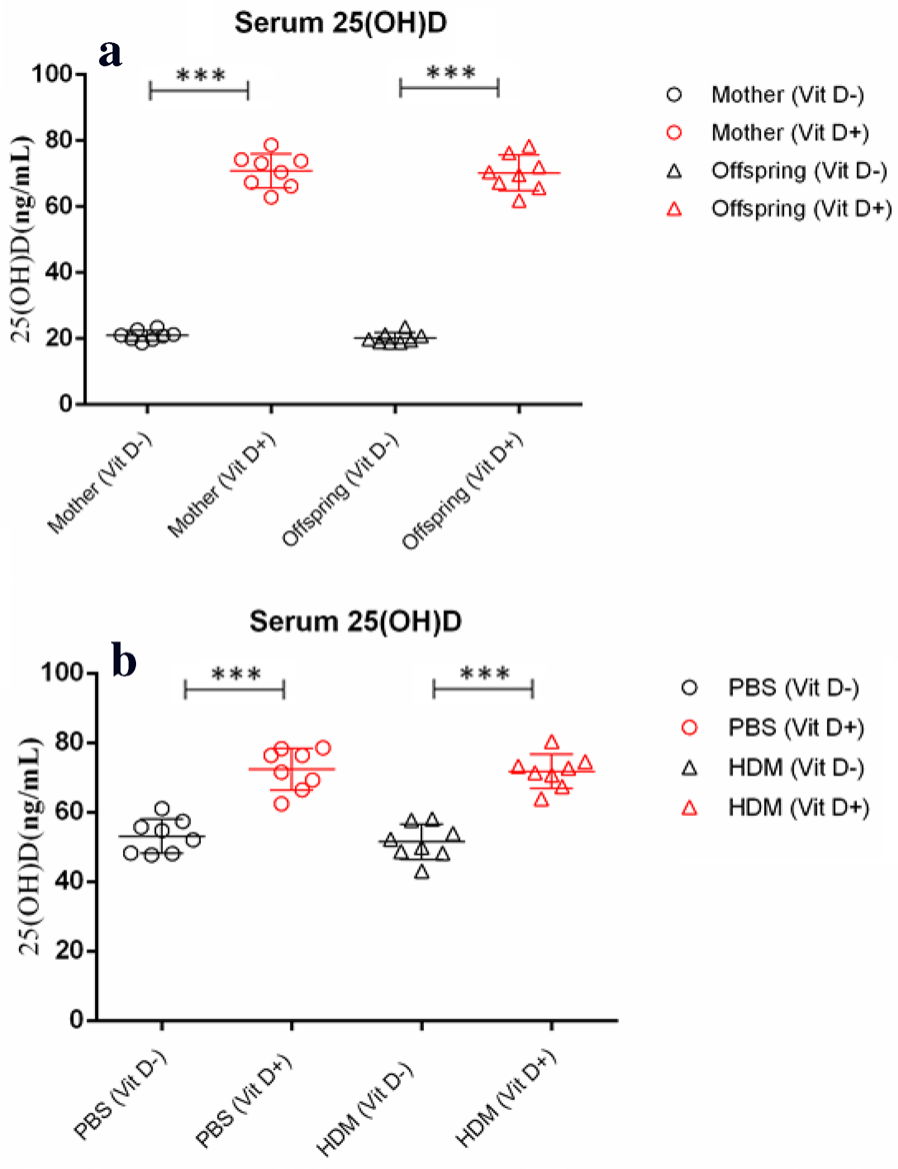
Maternal VitD deficiency reduces the levels of serum 25(OH) D in the mothers and offspring mice, regardless of inducing allergic airway inflammation. Female BALB/c mice at 4–6 weeks of age were randomized and fed with normal chow as the control or VitD deficient diet as the (VitD-) for 4 weeks before mating, throughout the gestational period and up to the weaning of their offspring mice. **a** The levels of serum 25(OH) D were measured. The offspring mice were randomized and injected with PBS or HDM to induce allergic airway inflammation. **b** The levels of serum 25(OH) D in individual mice were measured. Data are expressed as the mean values of individual mice in each group (n = 8) from three separate experiments. *p < 0.05, **p < 0.01, ***p < 0.001

**Fig. 3:**
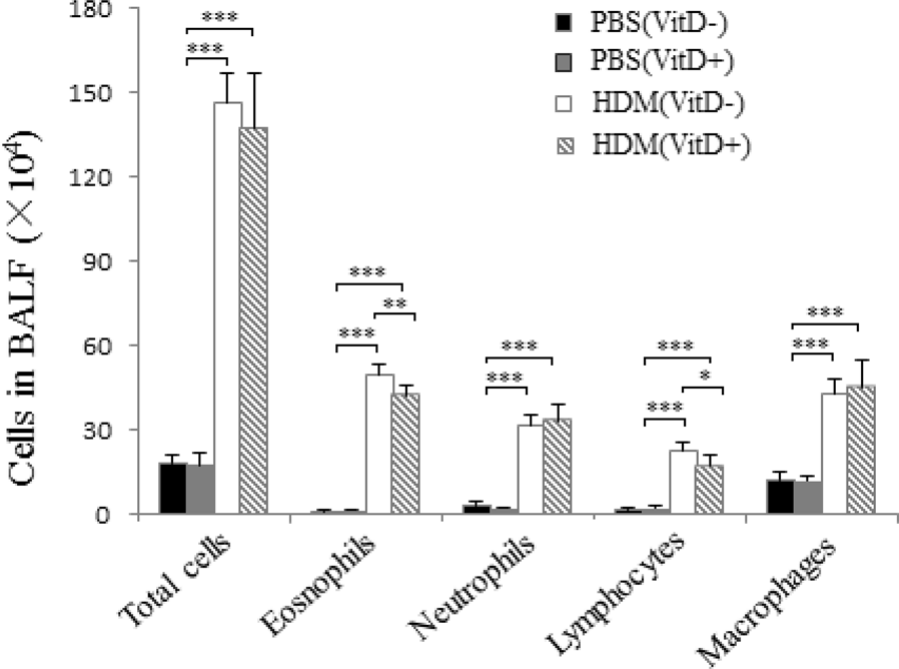
VitD deficiency increases the number of eosinophil and lymphocyte infiltrates in the lung of mice with HDM-induced allergic airway inflammation. Following inducing allergic airway inflammation by HDM injection, the numbers of different types of inflammatory infiltrates in in bronchoalveolar lavage fluid (BALF) samples were hematological stained and counted in a blinded manner. Data are shown as mean ± SD of each group (n = 6 mice per group) from three separate experiments, *p < 0.05, **p < 0.01 ***p < 0.001

To understand how low serum VitD3 levels in mothers affect their offspring following induction of allergic airway inflammation, female BALB/c mice were fed with normal chow or low VitD3 diet for 4 weeks and duration of pregnancy, and they remained the same kind of diet until the offspring weaning. Analysis of serum 25(OH) D revealed that the levels of serum 25(OH)D in the mothers with continual low VitD3 supply and their offspring mice were significantly lower than those with normal chow (p < 0.001, Fig. 2a). Following HDM-induced allergic airway inflammation, the levels of serum 25(OH)D in the PBS (Vit D-) and HDM (Vit D-) groups were similar, and significantly lower than those in the PBS (Vit D +) and HDM (Vit D +) groups of mice (p < 0.001, Fig. 2b). There was no significant difference in the levels of serum 25(OH)D between the PBS (Vit D +) and HDM (Vit D +) groups of mice (Fig. 2b). To understand the importance of VitD deficiency in the HDM-induced allergic airway inflammation, we collected BALF from individual mice and analyzed their inflammatory infiltrates. Although there was no significant difference in the numbers of total infiltrates, neutrophils and macrophages between the HDM (Vit D-) and HDM (Vit D +) groups of mice the numbers of eosinophils and lymphocytes in the HDM (Vit D-) were significantly greater than that in the HDM (Vit D +) group (p < 0.05, p < 0.01, Fig. 3). Such data indicated that feeding with low VitD3 diet in the mothers resulted in lower serum 25(OH)D in their offspring regardless of inducing allergic airway inflammation and increased eosinophil and lymphocyte infiltrates in the lung of offspring mice.

### Lower serum VitD modulates the transcription of VDR, VDBP, CYP24A1 and CYP27b1 mRNAs in the lung of mice

**Fig. 4:**
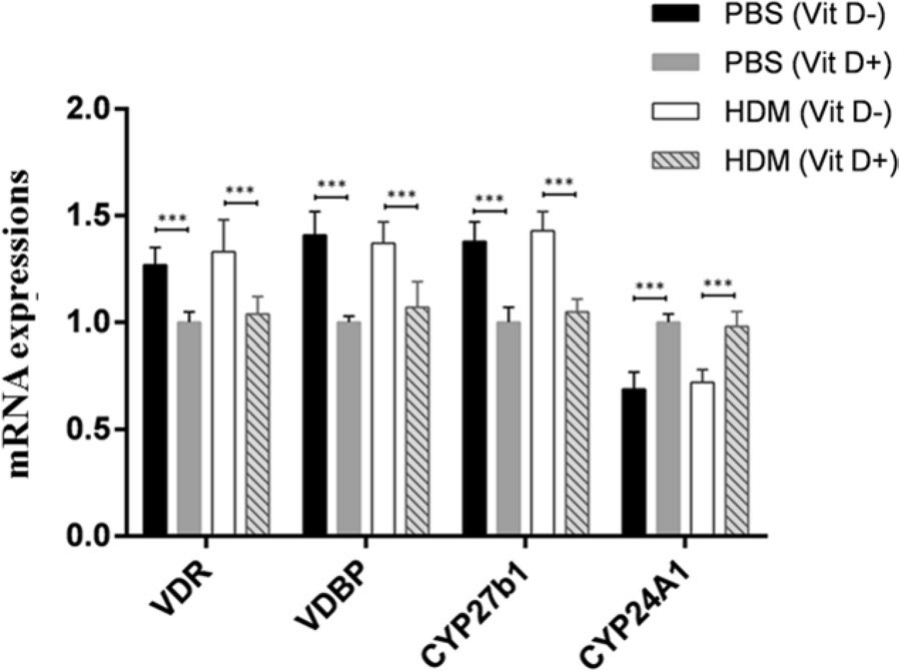
VitD deficiency modulates the transcription of VDR, VDBP, CYP27b1, and CYP24A1 mRNA in the lung of mice. The relative levels of VDR, VDBP, CYP27b1, and CYP24A1 mRNA transcripts to the control GAPDH in the lung tissues of individual mice were determined by quantitative RT-PCR. Data are expressed as the mean ± SD of each group (n = 8 mice per group) from three separate experiments. *p < 0.05, **p < 0.01, ***p < 0.001

Given that VitD3 is metabolized mainly by CYP27b1 to form active 1.25 (OH)_2_ D or degraded by CYP24A1 and its function depends on its receptor of VDR and VDBP-dependent intracellular transportation, we examined how lower serum 25(OH) D levels modulated the expression of VDR, VDBP, CYP27b1 and CYP24A1 in the lungs of different groups of mice by qRT-PCR. We found that the relative levels of VDR, VDBP and CYP27b1 mRNA transcripts in the lungs of the PBS (Vit D −) and HDM (Vit D −) groups were significantly higher than that in the PBS (VIt D +) and HDM (Vit D +) groups of mice (p < 0.001, Fig. 4). In contrast, the relative levels of CYP24A1 mRNA transcripts in the VitD deficient mice were significantly lower than those with normal VitD levels (p < 0.001, Fig. 4). There was no significant difference in the relative levels of these gene mRNA transcripts between the mice with PBS injection and those with HDM injection. Such data indicated that lower serum VitD levels modulated the transcription of VDR, VDBP, CYP24A1 and CYP27b1 mRNAs in the lung of mice, independent of inducing allergic airway inflammation in mice.

### VitD deficiency promotes inflammatory infiltration and decreases the frequency of regulatory cells in the lungs of the mice with allergic airway inflammation

**Fig. 5:**
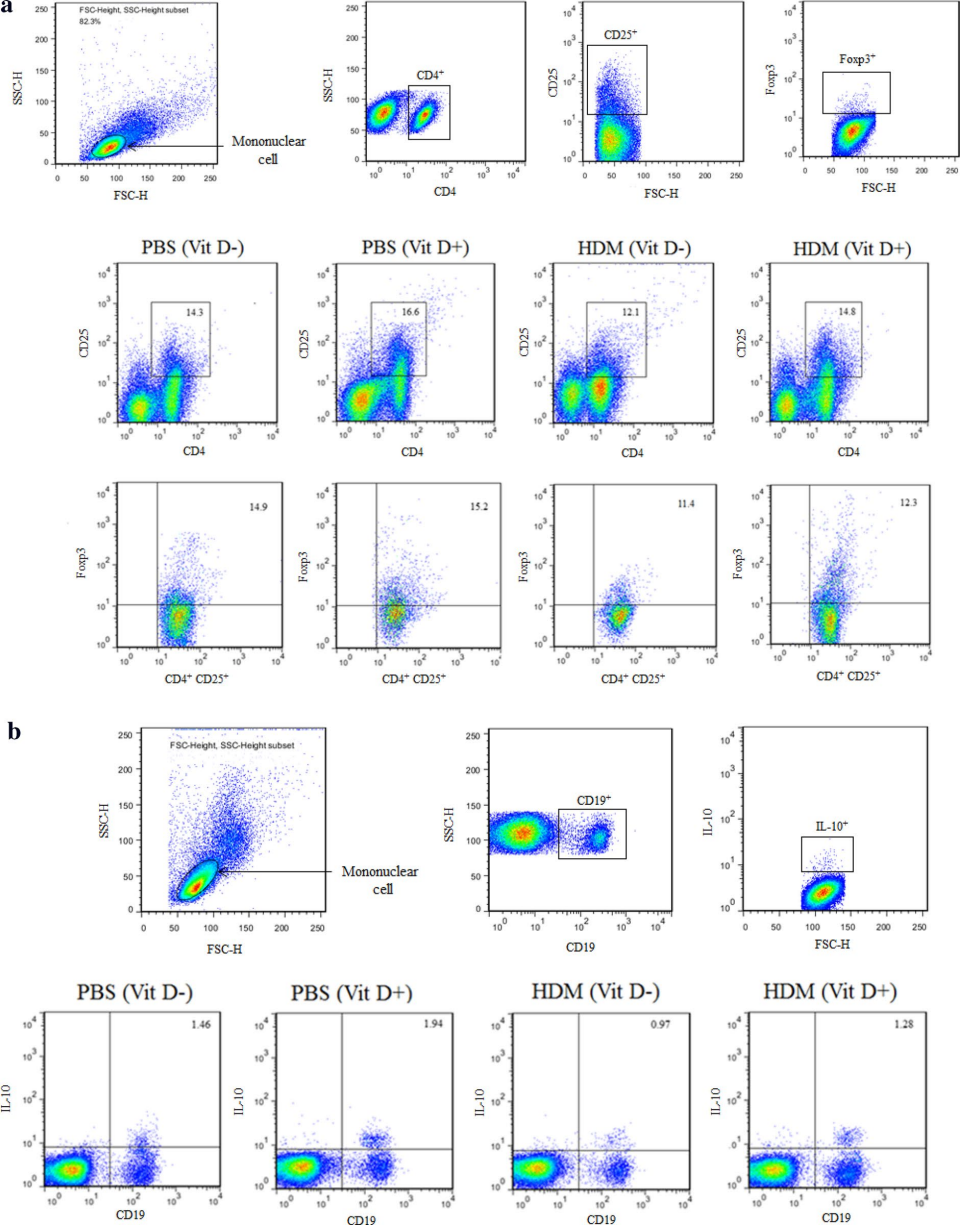

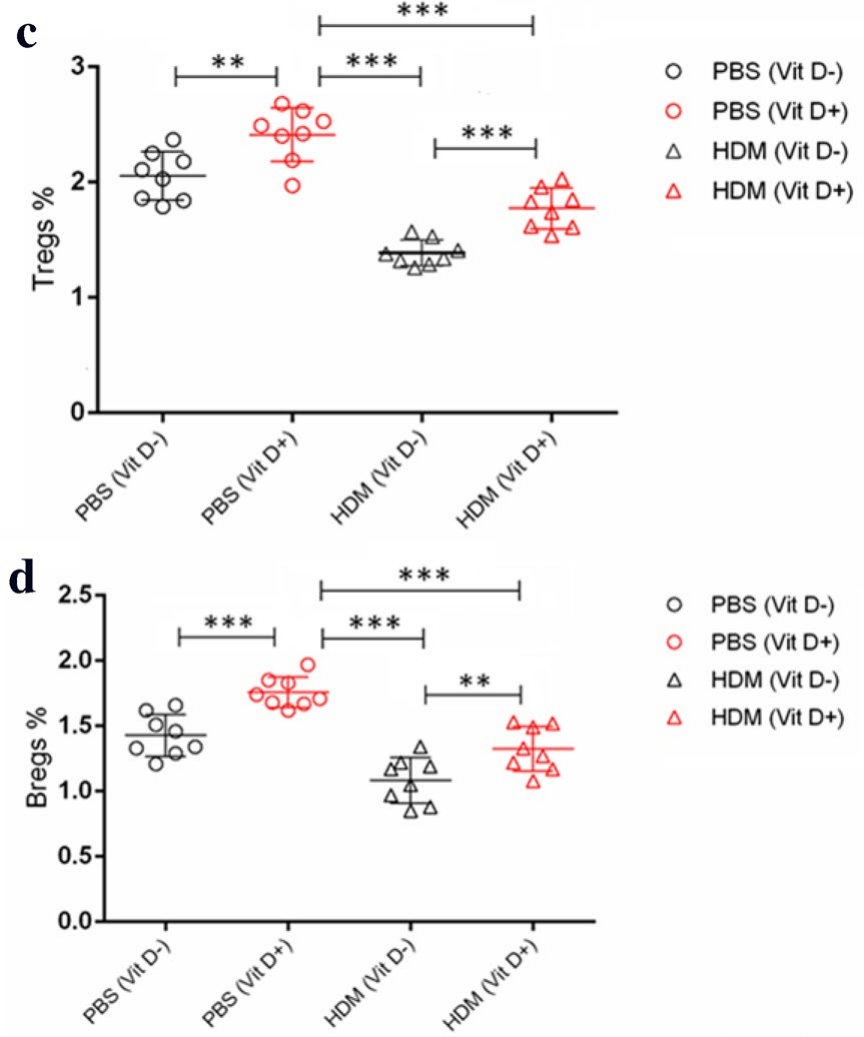
VitD deficiency reduces the frequency of Tregs and Bregs in the lungs of the mice. The offspring mice were randomized and injected with PBS or HDM to induce allergic airway inflammation. The percentages of CD4^+^CD25^+^Foxp3^+^ Tregs (**a**, **c**) and CD19^+^IL-10^+^ Bregs (**b**, **d**) in individual BALF samples were determined by flow cytometry. Data are representative flow cytometry charts and expressed as the mean values of individual mice in each group (n = 8 mice per group) from three separate experiments. *p < 0.05, **p < 0.01, ***p < 0.001

**Fig. 6:**
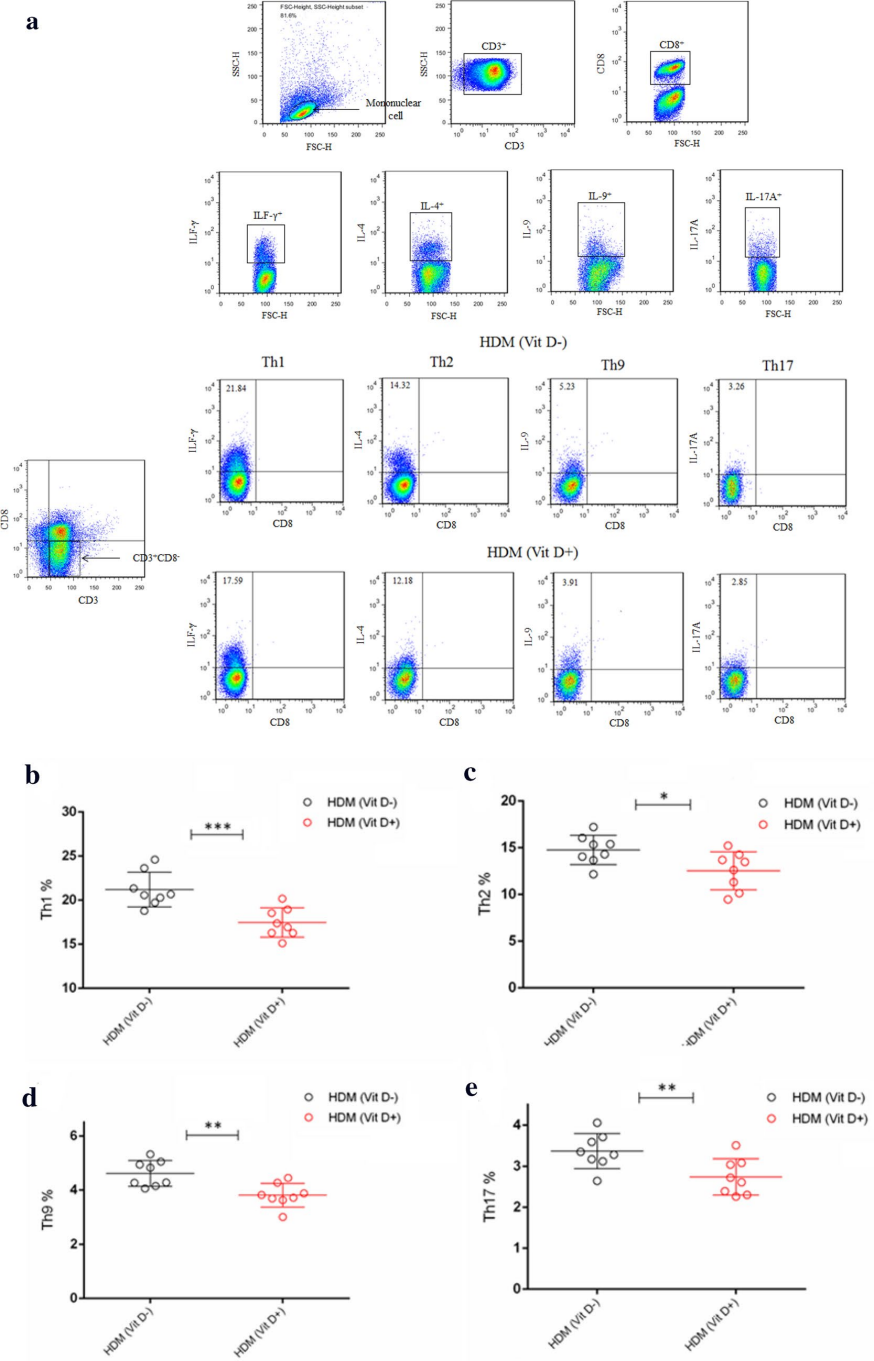
VitD deficiency increases inflammatory infiltrates in the lung of mice. The percentages of CD3 + CD4 + IFN-γ + Th1, CD3 + CD4 + IL-4 + Th2 cells, CD3 + CD4 + IL-9 + Th9 cells, and CD3 + CD4 + IL-17A + Th17 cells in the individual BALF samples were determined by flow cytometry. Data are representative flow cytometry charts and expressed as the mean values of individual mice in each group (n = 8 mice per group) from three separate experiments. **a** Representative flow cytometry charts. (**b**–**e**) Quantitative analysis. *p < 0.05, **p < 0.01, ***p < 0.001

To further understand the role of VitD deficiency in HDM-induced allergic airway inflammation, we characterized the frequency of different subsets of T cells in the BLAF of individual mice by flow cytometry. First, the frequency of Tregs and Bregs in the lung of the PBS (VitD +) group of mice were significantly higher than those in the PBS (VitD-) group (p < 0.01, p < 0.001, Fig. 5a–d). Similarly, the percentages of Tregs and Bregs in the lung of the HDM (VitD +) groups were also significantly higher than those of the HDM (VitD-) group (p < 0.001, p < 0.01), but significantly lower than that in the PBS (VitD +) group (p < 0.001 for both). Thus, the VitD deficiency decreased the frequency of Tregs and Bregs in the lung of mice, particularly in the mice with allergic airway inflammation. In comparison with that in the HDM (Vit D +) group of mice, significantly increased frequency of Th1, Th2, Th9 and Th17 cells were detected in the BALF samples of the HDM (Vit D-) group of mice (p < 0.05, p < 0.01, p < 0.001, Fig. 6). Therefore, VitD deficiency promoted allergic airway inflammation in the lungs of HDM mice by impairing Treg and Breg responses.

### Lower serum VitD enhances inflammatory cytokine responses in the lungs of the mcie with allergic airway inflammation

**Fig. 7:**
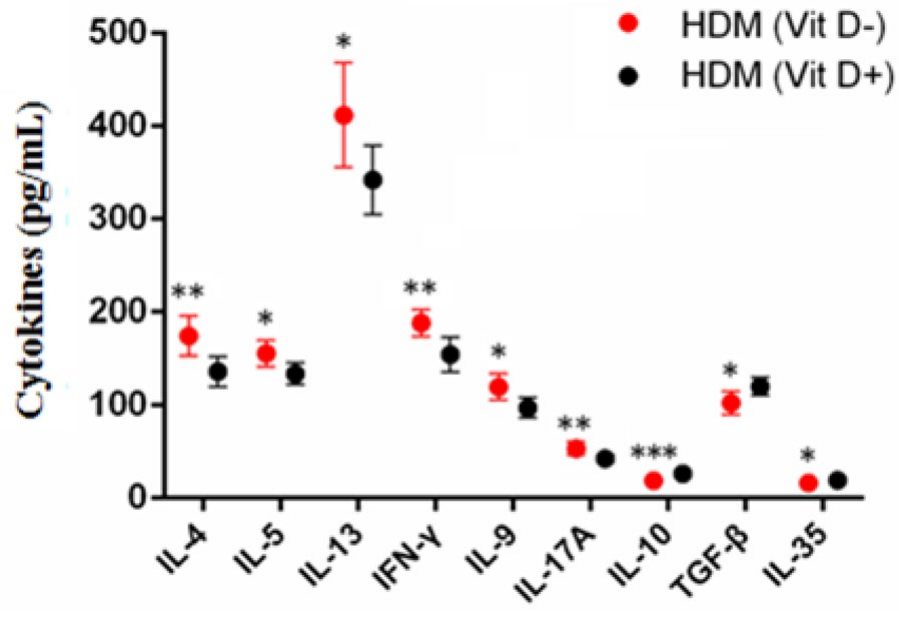
VitD deficiency modulates the levels of cytokines in the BALF from the mice with allergic airway inflammation. The concentrations of IL-4, IL-5, IL-13, IFN-γ, IL-9, IL-17A, IL-10, TGF-β1, and IL-35 in individual BALF samples were determined by ELISA. Data are expressed as the mean ± SD of each group (n = 6) from three separate experiments. *p < 0.05, **p < 0.01, ***p < 0.001

Induction of allergic airway inflammation can accumulate promote inflammatory infiltrates in the lung, where the inflammatory infiltrates can secrete cytokines. Analysis of cytokines in the BALF samples displayed that the levels of IL-4, IL-5, IL-9, IL-13, IL-17A and IFN-γ in the HDM (Vit D −) group were significantly higher than that in the HDM (Vit D +) group while the levels of IL-10, IL-35 and TGF-β1 were significantly lower than that in the HDM (Vit D +) group of mice (p < 0.05, p < 0.01, p < 0.001, Fig. 7). The increased levels of pro-inflammatory cytokines and decreased levels of inhibitory cytokines in the lungs suggest that VitD deficiency may induce the imbalance of pro-inflammatory and anti-inflammatory cytokine responses in the lungs, contributing to allergic airway inflammation in mice.

## Discussion

Previous studies have shown that VitD, VitE, folic acid and other vitamins can regulate allergic airway inflammation during the pathogenic process of allergic asthma [12–14], but the precise role of maternal VitD deficiency in offspring has not been clarified. Our data highlighted that maternal female mice with long-term lower VitD diet not only caused VitD deficiency in the recipients, but also induced VitD deficiency in their offspring even after fed with normal chow for couple weeks. Such data extended previous observations and support the notion that maternal VitD deficiency affects the offspring [7–9]. These data also suggest that maternal VitD deficiency may not only affect her own health, but may also have short-term and long-term adverse effects on her offspring's development and health.

VitD metabolism can epigenetically regulate gene expression during the fetal development, which may explain why VitD deficiency is associated with increased susceptibility to chronic diseases throughout life, such as autoimmune, metabolic, and infectious diseases [15, 16]. Previous studies have shown that maternal VitD deficiency increases the risk of asthma in the offspring [2, 5, 15]. Interestingly, we found that young mice with VitD deficiency had significantly higher levels of VDR, VDBP, CYP27b1, but lower CYP24A1 mRNA transcripts in the lung regardless of the development of allergic airway inflammation, relative to that in the mice with normal levels of VitD. The altered expression levels of VitD regulators may stem from compensative responses to defend against VitD deficiency in mice because VDBP functions to transport VitD, and CYP27b1 and CYP24A1 are crucial for maintenance of VitD levels [11, 17, 18]. It is possible that VitD deficiency may promote VDR, VDBP and CYP27b1 expression and down-regulate CYP24A1 expression to maintain serum 1.25(OH)_2_D3 levels and its function [19–24]. Such data also indicated that allergic airway inflammation did not affect the expression of these VitD regulators in mice. We are interested in further investigating how VitD deficiency regulates the expression of these VitD metabolic and functional regulators.

In this study, we found that VitD deficiency in young mice induced by maternal VitD deficiency deteriorated HDM-induced Th2-related allergic airway inflammation. Evidently, young VitD deficient mice with allergic airway inflammation had significantly greater lymphocyte and eosinophil infiltrates in their lungs. Those mice had significantly higher levels of IL-4, IL-5, IL-13, IL-9, IL-17A and IFN-γ, but lower levels of inhibitory IL-10, IL-35 and TGF-β1 in their BALF, indicating that VitD deficiency deteriorated the HDM-induced allergic airway inflammation in mice. Flow cytometry analyses revealed significantly higher frequency of Th1, Th2 and Th17 cells, but lower percentages of Th9 cells in the BALF of HDM mice with VitD deficiency. More importantly, significantly lower frequency of Tregs and Bregs was detected in the BALF of HDM mice with VitD deficiency. These novel data indicated that VitD deficiency altered the balance of pro-inflammatory T cell and regulatory cell responses to deteriorate the pathogenesis of allergic airway inflammation in mice. Hence, our findings may provide new insights into the pathogenesis of VitD deficiency-related allergic airway inflammation and theoretically, correction of VitD deficiency may be valuable in inhibiting the pathogenic process of allergic airway inflammation.

It is well known that activated CD4 + T cells can differentiate into IFNγ-secreting Th1, IL-4/IL-5/IL-13-secreting Th2, IL-17A-secreting Th17, IL-9-secreting Th9 and IL-10/TGFβ1-secreting Tregs, dependent on cytokines in the environment [2, 25]. In addition, CD4 + FoxP3 + nature Tregs exist in the body. HDM is one of the most significant environmental allergens and can induce potent Th2 responses and allergic airway inflammation [26–28]. In addition, allergen-specific Th1 and Th17 cells also contribute to the pathogenesis of allergic airway inflammation [29]. These inflammatory cells through their secreted cytokines promote eosinophil and neutrophil infiltration, activate mast cells, increase IgE production, stimulate mucus production and aggravate smooth muscle contraction [2, 30, 31]. Th9 can stimulate mucus secretion and increase the expression of Th2 cytokines. These inflammatory cells and their cytokines work together to eventually induce and exacerbate the HDM-induced allergic airway inflammatory [2]. In contrast, Tregs can effectively inhibit the pathogenic process of HDM-induced allergic airway inflammation by secreting inhibitory IL-10 and TGFβ1 [3, 25, 31]. Actually, our previous studies and those of others have shown that Tregs can inhibit the activation of effector T cells and their cytokine secretion, and reduce mast cell, eosinophil, basophil and neutrophil infiltration in the lung [25, 32]. Therefore, the imbalance of inflammatory cells and Tregs caused by VitD deficiency may be crucial for the development and progression of HDM-induced allergic airway inflammation.

We detected significantly lower levels of IL-35 in the HDM mice with VItD deficiency. Although IL-35 has been thought to be secreted by iTr35 cells, a unique kind of regulatory T cells, IL-35 can be also secreted by Tregs and Bregs. Functionally, IL-35 can effectively inhibit Th17 and Th1 responses and promote the proliferation of nTregs [33, 34]. The VitD deficiency may induce defect iTr35 development, contributing to impaired Treg and Breg responses during the development and progression of allergic airway inflammation.

CD19^+^IL-10^+^ Bregs are important for maintenance of peripheral tolerance and the deficiency in Breg responses contributes to the development and progression of some inflammatory autoimmune diseases [35, 36]. Functionally, Bregs can, through their secreting IL-10, inhibit inflammatory T cell responses in animal models of infection and autoimmune disease [37, 38]. Furthermore, Bregs can also promote Treg proliferation, and the functional transformation of CD4^+^CD25^−^ effector T cells into CD4^+^CD25^+^Foxp3^+^ Tregs [39]. In this study, we found that VitD deficiency not only decreased the frequency of Tregs, but also reduced the percentages of Bregs in the lungs of HDM mice although these mice were fed with normal chow during the induction of allergic airway inflammation. Although the decreased frequency of both Tregs and Bregs was moderate the decreased frequency of Tregs and Bregs may promote the expansion of inflammatory Th1, Th2, Th9 and Th17 cells in the lungs of HDM-injected mice with VitD deficiency, contributing to the development of airway inflammation. Such data also indicated that VitD supplement could correct VitD deficiency, but did not correct the imbalance of inflammatory and inhibitory responses during the pathogenic process of allergic airway inflammation in mice. Therefore, combination of VitD supplement with other reagents to inhibit inflammation and promote regulatory cell responses may be valuable in control of allergic airway inflammation in individuals with VitD deficiency.

## Conclusion

In summary, our data indicated that maternal VitD deficiency resulted in lower serum 25(OH)D in the offspring mice and following induction of HDM-based allergic airway inflammation, VitD deficiency significantly increased informatory lymphocyte and eosinophil infiltration, accompanied by elevating pro-inflammatory cytokines and reducing inhibitory cytokines in the lung of HDM-injected mice. VitD deficiency significantly enhanced pro-inflammatory Th1, Th2, Th9, Th17 cell responses, but attenuated Treg and Breg responses. Hence, VitD deficiency impaired inhibitory Treg and Breg responses, contributing to the pathogenesis of allergic airway inflammation in mice. Our findings may shed new lights in the mechanisms underlying the role of VitD deficiency in the development of allergic airway inflammation and uncover therapeutic targets for intervention of allergic airway inflammation.

## Data Availability

Not applicable.

## Abbreviations

VitD: Vitamin D
HDM: House dust mite
BALF: Bronchoalveolar lavage fluid
VDR: VitD receptor
VDBP: VitD-binding protein
Tregs: Regulatory T cells
Bregs: Regulatory B cells
NIH: The National Institutes of Health
ELISA: Enzyme-linked immunosorbent assay
APC: Allophycocyanin
FITC: Fluorescein isothiocyanate
PE: Phycoerythrin
